# A proposed paradigm shift in the management of distal radius fractures

**DOI:** 10.1016/j.jor.2023.11.065

**Published:** 2023-11-29

**Authors:** Panu H. Nordback, Tharun Ragupathi, Andre.E.J. Cheah

**Affiliations:** a Department of Hand and Reconstructive Microsurgery, National University Hospital, Singapore; b Musculoskeletal and Plastic Surgery, Department of Hand Surgery, University of Helsinki and Helsinki University Hospital, Finland

**Keywords:** Distal radius fracture, Corrective osteotomy, Wrist, Hand surgery, Orthopaedics, Injury

## Abstract

**Background and objective:**

Distal radius fractures represent a remarkable orthopaedic entity. Most distal radius fractures can be treated conservatively with closed reduction and immobilisation with satisfactory results, while open reduction and internal fixation is reserved for displaced fractures. Our objective was to propose a paradigm shift in the management of distal radius fractures.

**Methods:**

A literature search of management of distal radius fractures was conducted. PubMed and Cochrane databases were used for the search. English articles with open access or institutional subscription availability were included.

**Key content and finding:**

Current literature supports operative management for younger active patients with defined radiographic inclusion parameters, but among the elderly there is little evidence of benefit. Most orthopaedic literature defines “elderly” as patients above 65 years of age. Non-surgical treatment for fractures of the distal radius tends to yield satisfactory functional results, and these favourable outcomes do not necessarily align with normal radiological parameters. For the minority of patients that have symptomatic malunion, corrective osteotomy is a good option to improve the function provided the symptoms can be clearly attributed to the malalignment.

**Conclusion:**

The vast majority of distal radius fractures can be managed conservatively. Further studies are recommended to explore the feasibility of advocating for universal conservative treatment for patients with less functional demands while still having the option of staged surgery in the form of corrective osteotomy where there is symptomatic malunion amenable to anatomical correction. Future research should also aim to identify patients who would benefit most from surgical intervention by considering the type of functional recovery needed, rather than relying predominantly on the patient's chronological age as the determining factor in the decision-making process.

## 1 Introduction

In the adult population, distal radius fractures (DRFs) are the most common fractures, representing almost one-fifth of all fractures, and their incidence has been on an upward trend.^1^ It has been recently estimated that the incidence of distal radius fractures (DRF) ranges from 178.1 to 252.4 per 100,000-person years,^2^ with accidental falls on an outstretched hand forming the primary mechanism of injury for these fractures.^3^ The age distribution of patients is bimodal, with an initial peak among 18- to 25-year-olds, and a second peak among elderly patients in their sixties.^1^^,^^4^ Of note, postmenopausal osteoporosis increases the fracture risk in females fourfold as compared to males.^5^

Most DRFs can be treated conservatively with satisfactory results. Conservative management is achieved with a cast or splint immobilisation after attempted closed reduction, where warranted.^6^ Current evidence demonstrates that young and active patients with displaced DRFs and higher functional demands benefit from operative treatment.^7^^,^^8^ Open reduction with volar locked plating (VLP) has increasingly become the preferred surgical technique to treat DRFs and the incidence of operative treatment has also increased.^9^^,^^10^

Among most elderly patients, the prevailing evidence does not typically support operative treatment for DRFs.^7^^,^^10^ While a vast majority of patients regain good levels of function with conservative treatment, there are a minority of patients who experience debilitating residual symptoms. Ideally, surgeons would be able to discern between these groups, offering early surgery only to the latter and achieving the best risk-benefit ratio for both patients and the healthcare system. While scores have been used to help in this matter, many of them only predict malunion and not dysfunction.^11^ This is unsatisfactory given that we know that malunion does not always result in significant dysfunction.^11^^,^^12^ In practice, many surgeons are caught between the overuse of surgical treatment and the risks of complications that come with it. As such, we propose a paradigm shift in the management of DRFs in this group of patients – one that promotes universal conservative treatment as the first line for all DRFs in patients with low demand lifestyles, with corrective surgery being used only as a staged procedure in the minority of patients that report residual dysfunction after adequate rehabilitation.

## 2 Methods

**Fig. 1:**
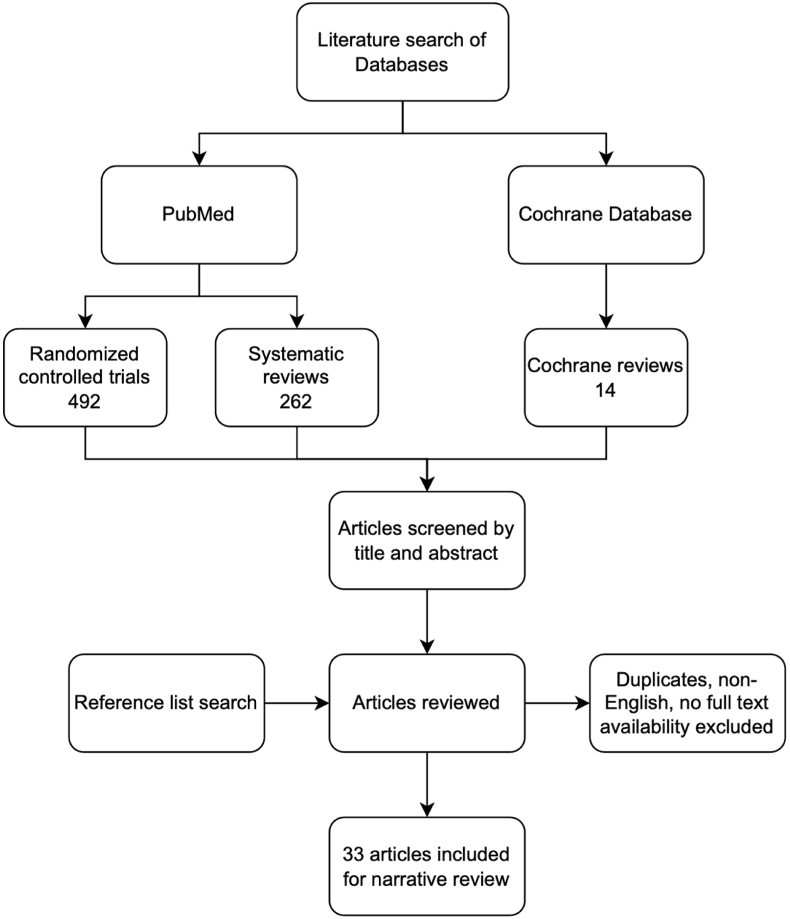
Flowchart of the conducted literature search.

The authors carried out an extensive literature search using PubMed and Cochrane Database, with a focus on randomized controlled trials and systematic reviews, including literature published up until the end of February 2023. For this narrative review, the scope was limited to articles written in English that were either freely accessible or available through institutional subscription (Fig. 1). The authors undertook the final selection process in a bid to find the answers to the following questions that would inform them of the evidence that might support their proposed paradigm shift:

1. Is it true that most elderly patients with DRFs treated conservatively have good function even in the setting of malunion?
2. In those who have malunion and dysfunction, what are the functional issues face?
3. For the patients with these types of functional issues in the setting of malunion, has corrective surgery been successful in achieving functional recovery?
4. Is there a group where we should avoid surgery even if functional problems occur in the setting of malunion?
5. Are there better alternatives to using chronological age as a factor when deciding if surgery for patients with DRFs – either early or staged – is suitable?

## 3 Discussion

### 3.1 Most elderly patients with DRFs treated conservatively have good function even in the setting of malunion

Evidence has progressively been leaning towards conservative management of DRFs in elderly populations, because of little proven clinical superiority, known hardware-based problems and similar outcomes at a minimum of 1 year follow-up when compared to operative management.^7^^,^^10^ It has also been demonstrated that patients over the age of 60 tolerate less favourable radiological values relatively well.^11^^,^^13^ As a result, radiological malunion in itself, at least within elderly populations, should no longer be considered a complication resulting from conservative treatment and the focus should be advocating for a management option that will deliver functional restoration while minimising risks associated with the chosen management option.

### 3.2 Ulnar-sided wrist pain and restriction of pronosupination are the main manifestations of dysfunction in the setting of malunion

Distal radioulnar joint (DRUJ) problems – including pain, restricted pronosupination and occasionally instability – are recognized potential issues after DRFs. Anatomically bony deformities, cartilage defects, the triangular fibrocartilage complex and the extensor carpi ulnaris tendon, including its sheath, are considered as the key culprits behind DRUJ problems.^14^ It has been estimated that bony architecture of a normal wrist accounts for approximately 20 % of DRUJ stability^15^ and intra-articular incongruency, and thus sigmoid notch malalignment or ulna-plus anatomy have been postulated as bony reasons for ulnar-sided wrist pain and limited pronosupination in DRF patients.^16^ Ulnar-sided wrist pathology has been reported to occur from 2 to 37 % of DRF cases^17^^,^^18^ and is interestingly also common after operatively treated DRFs.^19^ Admittedly, reduced pronosupination (particularly supination) can occur after DRFs due to the above-mentioned reasons, but most patients regain sufficient functional range of motion over time.^20^ There is no clear consensus on how best to address these issues and it is reasonable to start with standard conservative treatment strategies, such as activity modifications, splints or straps, analgesics and physiotherapy. A variety of surgical management options, depending on the aetiology, have been introduced over time without clear superiority of the available techniques.^20^

### 3.3 Corrective osteotomy and other salvage procedures are feasible options for patients with symptomatic radius malunion

**Fig. 2:**
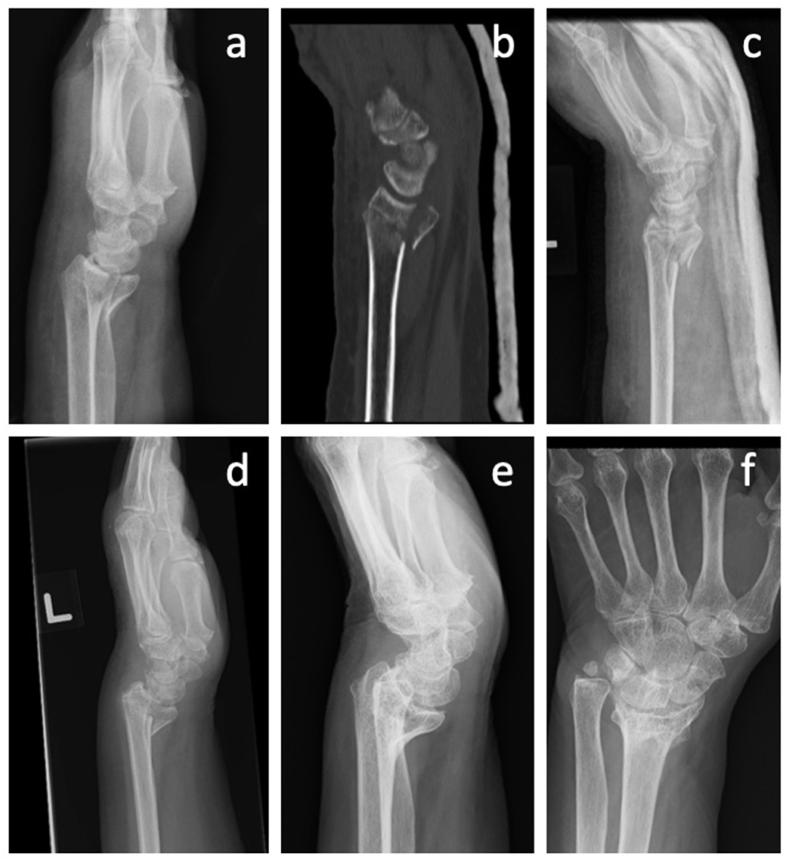
A 68 year old female sustained a closed distal radius fracture (a,b) that was managed conservatively by immobilisation (c). The fracture had healed in six weeks' time (d) and remodelled as shown by six months (e,f). The patient had some ulnar sided wrist pain, but her pronosupination was subjectively sufficient. Corrective osteotomy with ulnar head resection was discussed, but she was unwilling for further management as the symptoms were tolerable.

**Fig. 3:**
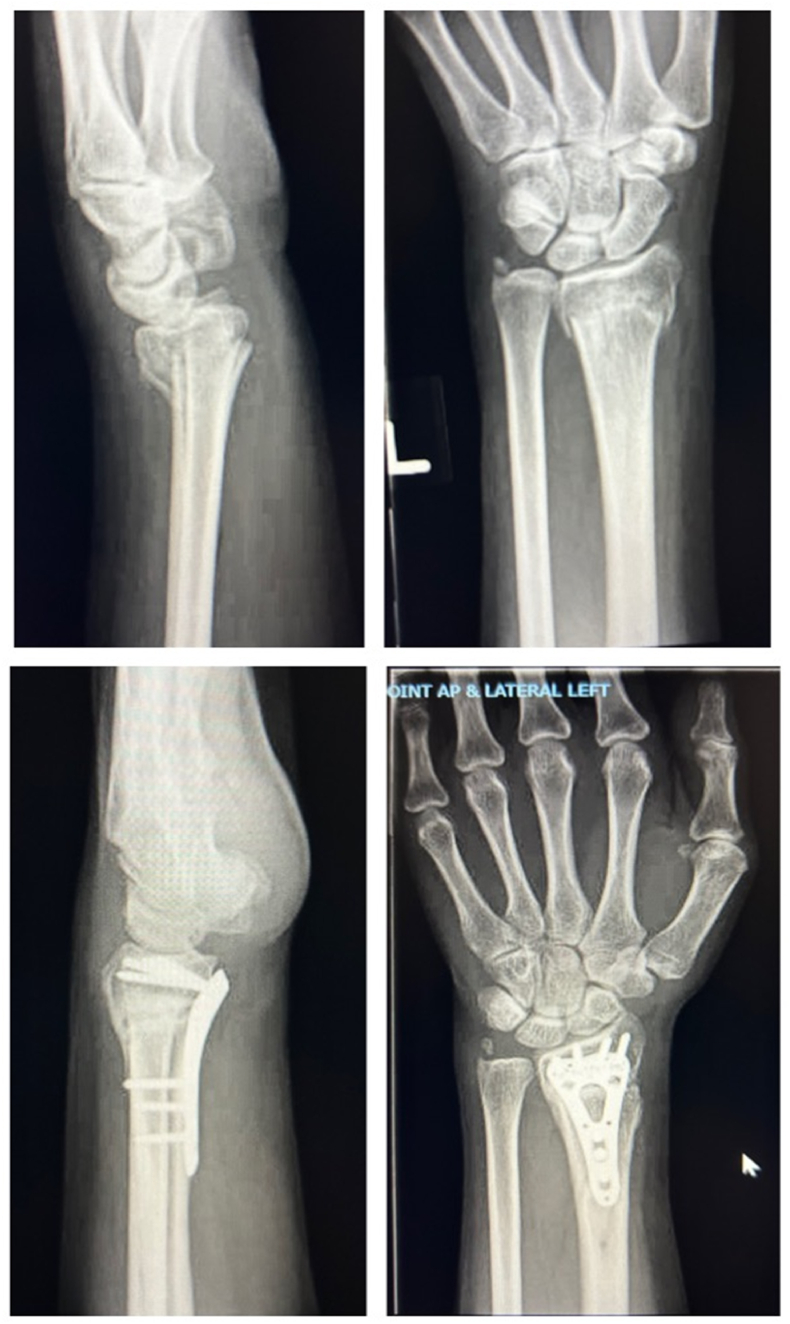
A 66-year-old female who sustained a distal radius fracture that was initially treated conservatively, but she had residual ulnar sided wrist pain that was successfully managed with a corrective osteotomy.

For patients with symptomatic malunion, corrective osteotomies have been shown to improve functional outcomes on long-term follow up across multiple studies.^21^^,^^22^ Although it is widely acknowledged that patients with symptomatic malunion should be considered for corrective osteotomies, there is currently no consensus regarding specific radiographic parameters that can reliably identify individuals who would derive the greatest benefit from these procedures.^21^^,^^23^ Common problems encountered are ulnar sided wrist pain and impaired pronosupination of the wrist (Fig. 2, Fig. 3) and it is the authors’ opinion that correction of the extra-articular indices of malunion are key to addressing these two problems. In patients where a corrective osteotomy is not possible, e.g. where there is significant osteoarthritis, salvage procedures may be considered. The primary objective and consideration of surgical salvage procedures for DRFs is to establish a stable and pain-free joint. Although complete restoration of wrist function is not always achievable through salvage surgery, it frequently succeeds in relieving pain and enabling a basic range of motion required for everyday tasks.^24^ Consequently, it is important to consider additional procedures such as ulnar shortening osteotomy (often performed alongside a distal radius corrective osteotomy), the Sauve-Kapandji procedure, and ulnar head resection, as important options to employ as the salvage procedure.^25^^,^^26^

### 3.4 Consider avoiding surgery in frail elderly patients even in the setting of symptomatic malunion

Defining “elderly” can be complex, as there is much heterogeneity in the definitions of elderly across literature. Orthopaedic research commonly defines an elderly population using chronological age alone, with almost 50 % of studies defining this population as those aged above 65 years old.^27^ However, using chronological age alone as a metric can be problematic. Life expectancy across the world has risen greatly in recent years, and this measure does not account for geographical variations in life expectancy or variations in level of function and independence.^28^ An “active” patient refers to one who possesses full independence in performing activities or daily living and actively engages in social, cultural, spiritual, and/or civic pursuits. Importantly, this state of being “active” is not necessarily related to one's chronological age. For this group, it becomes crucial to prioritize efforts in restoring a premorbid state of activity. Conversely, when dealing with frail patients, a different approach may be warranted.

Frail patients may be best objectively identified with measures such as frailty indices – these patients may be more prone to poor outcomes following surgery, on top of having high anaesthetic risk. Frailty indices have consistently performed better than chronological age at predicting poor outcomes^29^ and post-operative complications across disciplines. Frailty indices consider multiple factors that impact a patient's health and recovery, including physical function, cognitive function, and social support. Studies have shown that high frailty index scores are associated with an increased risk of post-operative complications in distal radius fractures.^30^ It may be beneficial to consider incorporating frailty indices to pre-operative assessment protocols (in addition to chronological age) when identifying patients who may be at higher risk for poor outcomes following surgery for distal radius fractures. This approach may provide a more comprehensive assessment of a patient's overall health and well-being and would allow surgeons to better identify patients who would be less likely to benefit from surgery, be it an early VLP or staged corrective osteotomy.

### 3.5 Patient specific functional scores may be an alternative to patient age when deciding if corrective surgery is indicated

Considering the idea that early surgical treatment aims to prevent malunion – which does not uniformly translate into dysfunction^31^ – the argument can be made to only recommend corrective surgery where malunion has led to dysfunction. Equally important is a surgeon's ability to identify specific features of malunion that contribute to a given patient's dysfunction and to have the confidence that a planned intervention can effectively correct the deformity and reverse the associated dysfunction. An illustrative example is seen in cases of DRFs with malunion causing ulnar-sided wrist pain in the presence of ulnar positivity, where surgical restoration of ulnar neutrality can alleviate pain (Fig. 2).

To facilitate discussions between the patient and surgeon, patient-specific functional scores can be highly beneficial. These scores assist patients in expressing their functional impairments effectively and help the surgeon focus on whether the planned anatomical correction directly addresses the dysfunction. Utilizing such scores promotes shared decision-making, aligns expectations for surgery, and enhances patient (and surgeon) satisfaction following successful anatomical correction of symptomatic malunion after conservative treatment.^31^, ^32^, ^33^

## 4 Conclusion

It is imperative for future research to prioritize the identification of patients who would derive the greatest benefit from early surgical intervention, versus those who would fare better with conservative treatment. Understanding the relative distribution of these two patient groups within the population with DRFs would provide valuable insights to surgeons, allowing for a more universal application of conservative treatment, where deemed rational. Ideally, in the authors' vision, early surgery would be reserved only for individuals with a high likelihood of developing symptomatic malunion, while staged corrective surgery would be offered to patients with malunion and specific functional scores that can be improved through surgical intervention. By adopting this approach, we can extend the benefits of conservative treatment to a larger proportion of DRF patients, while limiting the risks associated with surgery to those who genuinely require it.
